# Health-related quality of life in individuals with osteogenesis imperfecta in the United States: a cross-sectional study

**DOI:** 10.1186/s13023-025-04073-9

**Published:** 2025-10-23

**Authors:** Chloe E. Derocher, Jack E. Mulcrone, Erin M. Carter, Cathleen L. Raggio

**Affiliations:** Hospital for Special Surgery, 535 East 70th Street, New York, NY 10021 USA

**Keywords:** Osteogenesis imperfecta, Mental health, Pain, Grit, Patient-reported outcome measures

## Abstract

**Background:**

Osteogenesis imperfecta (OI) is a group of connective tissue disorders characterized by bone fragility and frequent fractures. The purpose of this study was to better understand the mental health burden, physical functioning, and health-related quality of life (HRQoL) in adults with OI in order to improve long-term care for this patient population.

**Methods:**

26 individuals with OI were enrolled into this cross-sectional study at the Hospital for Special Surgery in New York, NY and completed all questionnaires, which included demographics, medical history, depression (PHQ-8), anxiety (GAD-7), pain catastrophizing (PCS), activities of daily living (HAQ-DI), grit (12-item Grit Scale), and HRQoL (SF-36 and PROMIS-29).

**Results:**

11.5% of participants reported clinically significant symptoms of depression (PHQ-8 score ≥10) and 7.8% reported clinically significant symptoms of anxiety (GAD-7 ≥ 10). A prior psychiatric illness was reported by 57.7%. There was no difference in the level of pain catastrophizing in our cohort compared the general U.S. population (*p* = 0.363). Compared to the general population, individuals with OI had higher scores on the HAQ-DI (*p* < 0.001). Individuals with OI also reported significantly higher levels of resilience on the Grit Scale compared to the general population (*p* < 0.001). Individuals with OI had significantly lower scores on both HRQoL measures in terms of physical functioning domains (*p* < 0.001), but no significant difference compared to the general population across mental health domains.

**Conclusions:**

These findings suggest that individuals with OI may not necessarily experience a greater mental health burden compared to the general population despite lower scores in areas of physical functioning. We speculate this discrepancy between mental and physical health scores may be partially explained by their increased resilience. Altogether, a comprehensive approach to care that recognizes all aspects of living with OI, including pain, social functioning, and daily activities, is needed when caring for individuals with OI.

## Introduction

Osteogenesis imperfecta (OI), also known as “brittle bone disease,” is a group of hereditary connective tissue disorders characterized by increased bone fragility, resulting in bone deformities and frequent fractures across the individual’s lifespan [1]. Individuals with OI often exhibit extraskeletal manifestations of the disease, including blue sclera, short stature, cardiopulmonary complications, early-onset hearing loss, and dentinogenesis imperfecta [2]. The prevalence of OI is approximately 1 in 15,000–20,000 individuals [3]. It has been estimated that approximately 90% of cases are due to an autosomal dominant variant in the genes coding for type I procollagen (*COL1A1* and *COL1A2*), while the other ~10% of cases are mostly due to recessive variants in genes encoding proteins involved in the post-translational modification, folding, or transport of type I collagen [3]. Traditionally, OI has been classified under four main types based upon clinical presentation: type I (so-called mildest), type II (perinatally lethal), type III (most severe, survivable form of OI), and type IV (moderate) [4].

Current clinical care for individuals with OI is primarily focused on managing musculoskeletal problems and reducing fracture incidence. In a focus group-based study conducted by the Osteogenesis Imperfecta Foundation meant to address the adult OI community perspective, participants acknowledged the added burden on their mental health due to living with a constant fear of fractures and the stress of recurrent pain and hospital trips [5]. Authors concluded that the current approach to care for adults does not place adequate emphasis on the emotional well-being of the patients [5]. In addition, the current literature supports that many individuals with OI may be living with chronic pain, which can impact both physical and emotional wellbeing. In a study of 861 individuals with OI, including both adults and children, the prevalence of chronic pain was 41.8%, and the degree of pain interference was the same across OI types in adults [6]. Similarly, in a qualitative, interview-based study of 15 adults with OI, Shepherd and colleagues found that nearly all (14/15) experienced acute pain and most (10/15) reported living with chronic pain, consequently interfering with their sleep, mobility, and daily activities [7].

However, the existing literature seems to provide mixed conclusions in regard to the degree of psychological stress that individuals with OI might experience as a result of living with their diagnosis. The majority of studies currently available in the literature that have attempted to address quality of life in OI have only utilized one questionnaire, commonly the SF-36 [8, 9]. For instance, in a cohort of 322 adults with OI who completed the SF-36, Gooijer and colleagues found that physical functioning was significantly lower and bodily pain was significantly higher in adults with OI compared to the general population [9]. Participants with type I OI had significantly reduced mental health scores compared to the control group, but overall the study found that mental health domains were less affected than the physical domains in individuals with OI [9]. A study by Tosi and colleagues found that, based upon the PROMIS questionnaire, a group of 959 adults with OI had significantly lower mental health scores and higher depression and anxiety scores when compared to the general US population, although it was noted that the difference in mean scores did not qualify for clinical relevance [10]. In addition, a recent study by van Welzenis, et al. looked at the results of 1,440 adults with OI who completed the IMPACT survey, an international mixed-methods survey, and determined that living with OI strongly impacted patient-reported quality of life across physical, socioeconomic, and mental health domains. The authors also noted that the impact of OI on quality of life was closely related to factors such as self-reported OI severity and fracture frequency [11]. However, this study was not able to compare participant responses to the general population, nor did it utilize any validated quality of life outcome questionnaires.

The purpose of the present study was to conduct a thorough analysis of the health-related quality of life (HRQoL) of adults with OI by studying their responses to seven validated questionnaires covering their levels of pain and pain-catastrophizing, grit, depression and anxiety symptoms, physical function and disability, and overall physical and mental wellbeing. Taken together, we hope to use these measures to gain a more comprehensive understanding of the quality of life in this patient population, in order to monitor and improve care for those diagnosed with OI.

## Patients and methods

### Study design and participants

This was an IRB-approved prospective cross-sectional study focused on adults (18 years and older) with a clinical and/or genetic diagnosis of OI. Individuals with OI were enrolled during their standard-of-care visit to the Kathryn O. and Alan C. Greenberg Center for Skeletal Dysplasias at Hospital for Special Surgery between May 2021 and October 2023. This cohort was collected as part of a larger multi-center study which looked at health-related quality of life in individuals with skeletal dysplasias (excluding OI) [12]. The current study uses the same study protocol and methods as reported in Fagereng et al., except for the inclusion of the 12-item Grit Scale which we used to assess resilience in the OI cohort.

### Methods

#### Measures

Seven self-administered questionnaires were completed by all subjects in order to comprehensively assess their quality of life, emotional distress, and pain levels. These questionnaires were the RAND 36-Item Short Form Health Survey (SF-36), the Patient-Reported Outcomes Measurement Information System 29 + 2 Profile v2.1 (PROMIS-29), the Pain Catastrophizing Scale (PCS), the seven-item Generalized Anxiety Disorder questionnaire (GAD-7), the Patient Health Questionnaire Depression Scale (PHQ-8), the Stanford Health Assessment Questionnaire Disability Index (HAQ-DI), and the 12-item Grit Scale.

The RAND SF-36 is one of the most widely used overall health-related quality of life surveys in the world [13] and was used to assess participants’ general physical and mental functioning. This survey consists of 36 questions which encompass 8 health concepts: physical functioning, limitations due to physical health, pain, general health, energy/fatigue (vitality), social functioning, limitations due to emotional problems, and emotional well-being (mental health). Scale and summary scores were calculated from participant responses to the surveys. The eight scale scores included physical functioning, role limitations due to physical health, pain, general health, energy/fatigue, social functioning, role limitations due to emotional problems, and emotional well-being/mental health. The SF-36 has been widely studied and cited in other studies of health-related quality of life in people with skeletal dysplasias [9, 14, 15–23]. PROMIS-29 has been shown to be a valid measure with good psychometric properties [24] and was used to measure patient-reported outcomes that have major effects on quality-of-life, such as physical functioning, emotional distress, and social role participation in different patient populations [25]. The survey consists of 29 questions which cover 7 health domains: physical function, pain interference, fatigue, sleep disturbance, depression, anxiety, and ability to participate in social roles and activities. Inclusion of responses to both the SF-36 and PROMIS-29 in the same cohort will benefit future studies using either questionnaire [12]. Other studies have compared PROMIS-29 and SF-36 scores in cohorts of people living with musculoskeletal problems such as chronic pain, osteoarthritis, and low back pain [26, 27]. We used both of these valid and precise measures to assess important health domains which may impact QoL for patients living with rare disorders such as OI.

Participants also completed the Pain Catastrophizing Scale (PCS), which is used to evaluate the extent of catastrophic thinking that they have when they are in pain [28]. It consists of 12 statements that describe different thoughts and feelings that they may associate with pain, such as “I worry all the time about whether the pain will end,” “It’s awful and I feel that it overwhelms me,” “I keep thinking about how much it hurts,” and “I wonder whether something serious may happen.” The individual uses a four-point scale to indicate the degree to which they agree with the statements; the sum of these scores is the participant’s measure on the PCS. It has been established that a score ≥30 on the PCS indicates a clinically-relevant level of pain-catastrophizing [28].

Severity of anxiety among participants was measured using the Seven-Item Generalized Anxiety Disorder Questionnaire (GAD-7) [29]. The GAD-7 presents seven questions related to symptoms of anxiety; the participant indicates how often they have experienced these symptoms in the last two weeks. A total score of ≥ 10 on the GAD-7 was used as a cutoff for a clinically-relevant level of anxiety [29]. Similarly, depression symptoms among participants were measured using the Patient Health Questionnaire Depression Scale (PHQ-8) [30]. This questionnaire covers the first eight diagnostic criteria of depressive disorder in the Diagnostic and Statistical Manual of Mental Disorders IV (DSM-IV); the participant is asked to report how often they have experienced these symptoms in the last 2 weeks. A score ≥10 on the PHQ-8 was used as a cutoff for a clinically-relevant level of depression [30].

The HAQ-DI (HAQ Disability Index) was used to assess the participants’ functional ability as measured by their reported difficulty in accomplishing activities of daily living such as dressing, arising, eating, walking, hygiene, reach, grip, and other daily tasks (errands, chores, driving) [31].

The level of resilience or “grit” of the participant was measured using the 12-Item Grit Scale, which consists of 12 statements such as “I have overcome setbacks to conquer an important challenge,” “My interests change from year to year,” “I am a hard worker,” and “I finish whatever I begin.” The maximum score on this scale is 5 (extremely gritty) and the lowest score is 1 (not at all gritty) [32].

Finally, participants were also asked to complete a data collection sheet to assess demographics and other factors that may influence their quality of life, such as their highest education level, employment status, past surgical history, psychiatric history, and whether they use an assistive device. In addition, participants were asked on the data collection sheet to indicate the bodily locations in which they had experienced pain in the last four weeks and to rate the intensity of their pain on a scale from 1 to 10.

#### Data collection and analysis

Study data was collected and managed using REDCap electronic data capture tools hosted at the Hospital for Special Surgery [33, 34]. Statistical analysis was conducted using SPSS 29 for Windows (IBM Corp., Armonk, NY, USA). Mean OI questionnaire scores were compared to the general population via two-sided, one-sample t-tests and between types of OI via one-way ANOVA. Nonparametric binomial tests were used to compare proportions of the OI population above PHQ-8 and GAD-7 cutoffs compared to the general population. For all analysis, significance was designated by *p* < 0.05.

## Results

A total of 26 participants (23 female, 3 male) with osteogenesis imperfecta were enrolled in the study and completed all questionnaires. Participant characteristics are displayed in Table 1. The mean age of participants was 44.8 years, with 53.8% having completed higher education (a master’s or doctorate degree) and 57.7% employed in a full-time position. The average number of surgeries per participant was 8.88, with the most common type of surgery being upper and/or lower extremity surgery not otherwise listed on the collection sheet (38.5%).

**Table 1:** Sociodemographic characteristics of the participants (*n* = 26)

	Frequency (%)	Mean (Range)
**Female/male**	23 (80.8%) /3 (11.5%)	
**Age**		44.8 (22–73)
**Education level**^**a**^		
Primary	3 (11.5%)	
Secondary	9 (34.6%)	
Higher Education	14 (53.8%)	
**Employment Status**		
Full time	15 (57.7%)	
Part time	4 (15.4%)	
Full disability benefit	0 (0%)	
Other^b^	7 (26.9%)	
**OI type**		
Type I	13 (50%)	
Type III	2 (7.7%)	
Type IV	11 (42.3%)	
**Surgical burden**		
Total number surgeries		8.9 (0–30)
**Surgery type**		
Spine surgery	4 (15.4%)	
Ear surgery	4 (15.4%)	
Tonsillectomy	6 (23.1%)	
Foot surgery	8 (30.8%)	
Hand surgery	5 (19.2%)	
Joint replacement	4 (15.4%)	
Rodding	11 (42.3%)	
Leg realignment	3 (11.5%)	
Leg lengthening	1 (3.8%)	
Other upper extremity surgery	10 (38.5%)	
Other lower extremity surgery	10 (38.5%)	
Other	6 (23.1%)	

^a^Primary education defined as a highschool diploma or its equivalent, secondary education defined as a Bachelor’s or Associate’s degree, and higher education defined as a Master’s or doctorate degree

^b^Unemployed, retired, or student

Table 2 displays variables related to pain, grit, and ability to participate in activities of daily living compared to the general population. The mean self-reported pain intensity (on a scale from 1 to 10) experienced by participants over the last four weeks was 4.0 ± 2.6, and the most common locations of pain included the lower extremities (46.2%), lower back (34.6%), and neck (30.8%). However, the mean score on the Pain Catastrophizing Scale (PCS) was 11.5 ± 13.7, and only 3 (11.5%) participants met the threshold for clinically-significant levels of pain catastrophizing. There was no significant difference in mean PCS score between the OI participants and the general population (*p* = 0.393). OI participants scored higher on average on the 12-item Grit scale than the general population (3.86 ± 0.53 vs. 3.41 ± 0.67; *p* < 0.001). No significant differences were found in the mean grit score between men and women or between types of OI. OI participants also scored higher on the HAQ disability index than the general population (0.75 ± 0.78 vs. 0.25 ± 0.60; *p* < 0.001), and those with the most severe form of OI (type III) had a higher mean score on the disability index than types I or IV (2.06 ± 0.62 vs. 0.59 ± 0.70 and 0.72 ± 0.72 respectively; *p* < 0.001), which is consistent with previous literature [38].

**Table 2:** Pain, grit, and activities of daily living compared to the general population

	OI participants	General population	*p*-value
	Mean (SD)	Mean (SD)	
**Pain**			
Pain intensity	4.0 (2.6)		
Pain catastrophizing score	11.5 (13.7)	13.9 (10.1) [35]	0.393 (n.s.)
Above cutoff (≥30), n (%)	3 (11.5%)		
Pain site location^a^			
Headache, n (%)	2 (7.7%)	22.4%	
Neck, n (%)	8 (30.8%)		
Upper extremities, n (%)	5 (19.2%)	30.7%	
Upper back, n (%)	5 (19.2%)	39.0%	
Lower back, n (%)	9 (34.6%)		
Lower extremities, n (%)	12 (46.2%)	36.5%	
**Grit**			
Grit scale mean score	3.86 (0.53)	3.41 (0.67) [32]	< 0.001
**Activities of Daily Living**			
HAQ-DI total mean score	0.75 (0.78)	0.25 (0.60) [36]	< 0.001
Mean for type I OI	0.59 (0.70)		
Mean for type III OI	2.06 (0.62)		
Mean for type IV OI	0.72 (0.72)		

^a^Percentages of headache, upper and lower limb pain, and back pain in the U.S. population in 2019 are from NCHS Data Brief No. 415 [37]. Total percentages > 100% as participants may indicate pain in multiple locations

When assessing psychological and emotional distress, we found that the mean PHQ-8 score was 3.96 ± 3.91 and the mean GAD-7 score was 3.65 ± 3.33 (Table 3). The percentage of participants with clinically-relevant depression was 11.5% compared to 8.9% in the general population; similarly, the percentage of participants with clinically-relevant anxiety was 7.8% compared to 5.9% in the general population. These differences were not found to be significant. Just over half of respondents indicated they had at least one prior psychiatric diagnosis (15/26, 57.7%). The most commonly reported past psychiatric diagnosis within the cohort was anxiety (42.3%), followed by ADHD (34.6%). It is important to note that the two individuals whose scores on the GAD-7 exceeded the clinical cutoff were also ones who reported a history of anxiety on the data collection sheet. Conversely, two out of the three individuals who screened positive for clinically-relevant depression symptoms during their center visit (based on their PHQ-8 score) had not reported a previous history of depression.

**Table 3:** Measurements of physical and psychological distress among participants

	OI participants (n = 26)	General population	*p*-value
	Mean (SD)	Mean (SD)	
**Depression (PHQ-8 total score)**	3.96 (3.91)		
Above clinical cutoff (≥10), n (%)	3 (11.5%)	8.6% [30]	0.390 (n.s.)
**Anxiety (GAD-7 total score)**	3.65 (3.33)		
Above clinical cutoff (≥10), n (%)	2 (7.8%)	5.9% [39]	0.459 (n.s.)
**History of psychiatric diagnosis**			
Depression, n (%)	3 (11.5%)		
Bipolar disorder, n (%)	1 (3.8%)		
Anxiety, n (%)	11 (42.3%)		
ADHD, n (%)	9 (34.6%)		
OCD, n (%)	0 (0%)		
Eating disorder, n (%)	0 (0%)		
Schizophrenia, n (%)	0 (0%)		
PTSD, n (%)	3 (11.5%)		
Substance abuse, n (%)	1 (3.8%)		
Other, n (%)	1 (3.8%)		

**Fig. 1:**
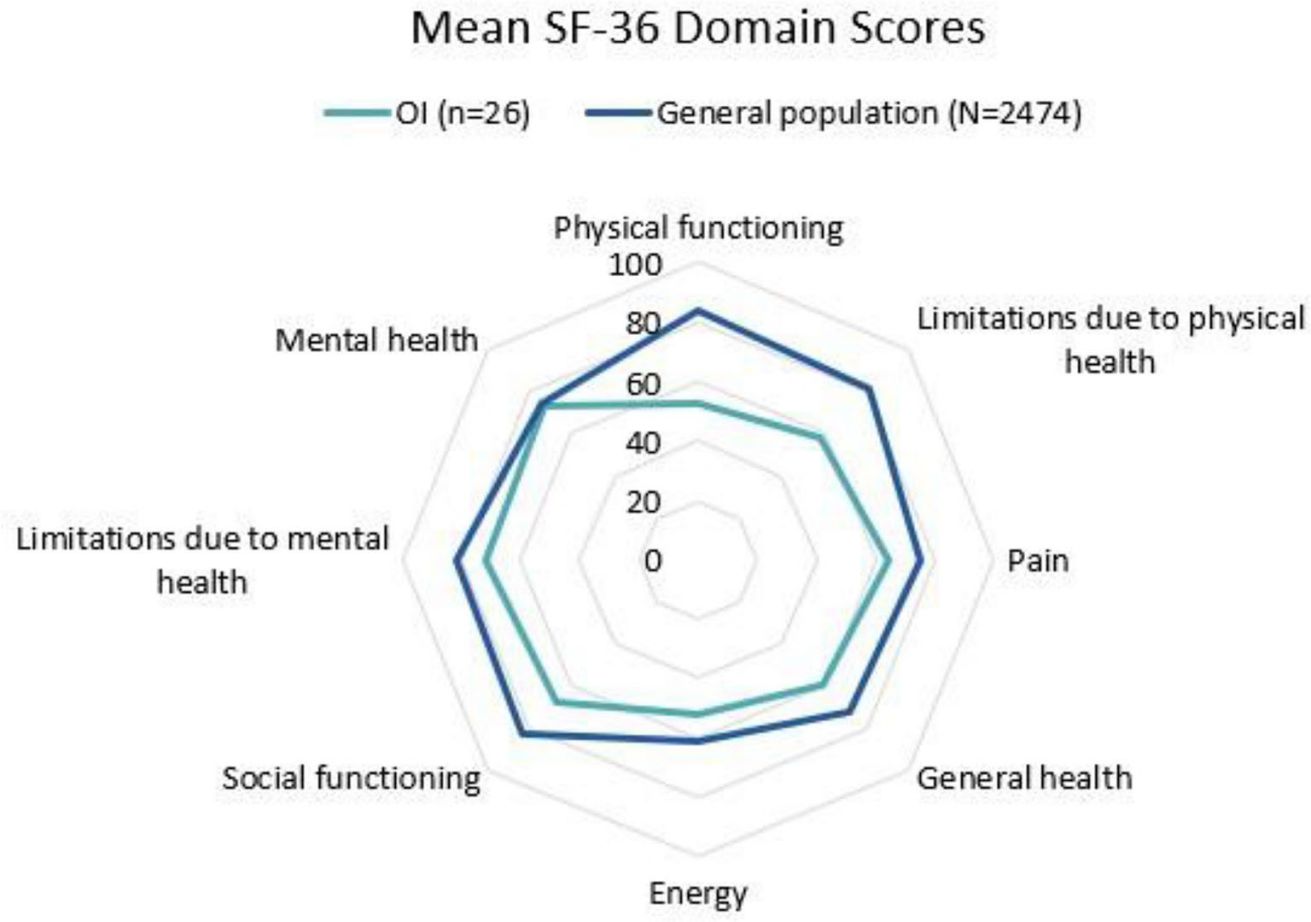
Mean SF-36 domain scores in the OI cohort compared to the general population

The SF-36 and PROMIS-29 were used to assess HRQoL (Table 4). Participants scored significantly lower in all of the SF-36 domains compared to the general U.S. population except for mental health, limitations due to mental health, and energy (*p* < 0.01). The domains that differed the most from the general population were physical functioning, social functioning, and limitations due to physical health (Fig. 1). On the PROMIS-29, OI participants reported a lower degree of physical functioning (mean score 41.2 ± 10.9; *p* < 0.001) in comparison to the U.S. general population. There was no significant difference in the participants’ scores on the depression or anxiety domains (mean scores of the PROMIS-29 compared to the U.S. general population), which was consistent with the mental health results of the SF-36 as well.

**Table 4:** General HRQoL measures compared to the general population

	OI participants (n = 26)	General population	*p*-value
	Mean (SD)	Mean (SD)	
**SF-36**^**a**^			
Mental health	73.2 (15.4)	74.7 (18.1)	0.622 (n.s.)
Limitations due to mental health	71.8 (40.8)	81.3 (33.0)	0.248 (n.s.)
Social functioning	67.8 (23.9)	83.3 (22.7)	0.003
Energy	51.7 (25.3)	60.9 (21.0)	0.077 (n.s.)
General health	59.2 (22.9)	72.0 (20.3)	0.009
Pain	64.1 (25.9)	75.2 (23.7)	0.040
Physical functioning	52.5 (33.2)	84.2 (23.3)	< 0.001
Limitations due to physical health	58.65 (43.0)	81.0 (34.0)	< 0.001
**PROMIS-29**^**b**^			
Physical function	41.2 (10.9)	50 (10)	< 0.001
Depression	50.9 (9.5)	50 (10)	0.770 (n.s.)
Anxiety	49.4 (9.8)	50 (10)	0.618 (n.s.)
Fatigue	48.75 (12.0)	50 (10)	0.601 (n.s.)
Sleep disturbance	51.0 (9.2)	50 (10)	0.588 (n.s.)
Social participation	52.1 (10.0)	50 (10)	0.289 (n.s.)
Pain interference	52.2 (9.5)	50 (10)	0.249 (n.s.)

^a^Mean SF-36 scores for the general U.S. population taken from Ware, et al. (1993), Farivar, et al. (2007) [40, 41]

^b^General population mean was set to a T-score of 50 with a SD of 10 [42]

## Discussion

Osteogenesis imperfecta (OI) is an inherited type I collagenopathy resulting in disordered connective tissue, often characterized clinically by increased bone fragility and frequent fractures [1]. Only a handful of studies have been previously conducted to better understand the quality of life of individuals living with OI, and these studies often focus on a single HRQoL outcome tool [8–11]. This cross-sectional study aimed to investigate the health-related quality of life of adults with OI, including aspects such as the prevalence of mental health symptoms, physical function and ability to participate in activities of daily living, and levels of grit and pain catastrophizing within our cohort.

In this study, we observed that clinically-significant anxiety and depression in the OI cohort were not statistically different than in the general population. Interestingly, while almost half of the participants reported having a previously-diagnosed history of anxiety, only two participants met the cutoff for clinically-relevant anxiety based on the GAD-7 questionnaire. This discrepancy may be explained by the fact that some participants may be treating their anxiety symptoms at the time of taking the surveys through medication or therapy and therefore report lower GAD-7 scores. We also observed that two of the individuals who reported current clinical symptoms of depression on the PHQ-8 at the time of the study did not report having a prior history of depression diagnosed by a psychiatrist. In addition, only one of the three individuals who reported having a prior history of depression screened above the clinical cutoff on the PHQ-8, which may further highlight the importance of successful intervention and treatment for individuals who struggle with their mental health.

The results of the SF-36, PROMIS, and HAQ-DI questionnaires illustrated that participants with OI face significant challenges in regard to their physical functioning and health, as seen from the significantly lower scores in these domains compared to the general population. However, our findings indicate that participants with OI do not necessarily score lower on the SF-36 and PROMIS-29 mental health-related domains. The results of the mental health component of the SF-36 are consistent with previous studies on HRQoL in OI, which have demonstrated that individuals with OI tend to score lower on the physical component of the questionnaire but not the mental component. In a study of a similar size conducted over two decades ago, Widmann, et al. (2002) showed that individuals with OI had significantly lower scores in physical functioning, limitations due to physical functioning, and bodily pain, but not in any of the other categories [8]. It is notable that these findings of mental health resiliency, even with a higher physical health burden, seem to have remained consistent over time. Similarly, a study by Murali et al. found that the mean scores on the mental component of the SF-12 (a shortened version of the SF-36) were not significantly different from the U.S. general population for those with type I and IV OI [43]. When examining the other measures we utilized such as the PROMIS-29, the GAD-7, and the PHQ-8, our results similarly align with these authors’ conclusions; these different measures all indicated that the cohort did not experience higher levels of anxiety or depression than the general population. The results of van Welzenis and colleagues’ work looking at data from the IMPACT survey found that 66% of the adults with OI reported that OI impacts their mental health, with 30% indicating a “moderate-to severe” impact [11]. This study also noted that mental health concerns were highest among those with OI who had experienced vertebral or other fractures in the past year, and that quality of life was driven by factors such as self-perceived OI severity and frequency of fractures [11].

The available literature suggests that individuals with OI may have a milder mental health impact compared to other skeletal dysplasias. When comparing our results to previous work by Yonko, et al. looking at people with achondroplasia, fewer individuals with OI reported a past psychiatric diagnosis of depression, and individuals with OI also scored higher on the mental health components of the SF-36 [14]. In addition, when looking at the previously-published HRQoL data from this study by Fagereng et al., which investigated quality of life in a cohort of other skeletal dysplasias, excluding OI, across three countries using these same methods, we see that those with rarer forms of skeletal dysplasias (grouped as “other SD”) appear to have a much higher overall mental health burden compared to those with OI [12].

Importantly, we found that the individuals with OI had a significantly higher score on the 12-item Grit Scale compared to the general population. Interestingly, grit did not vary by sex or by OI type, a factor which should correspond to clinical severity. This is the first study examining grit in any rare disease population. Future work should investigate what characteristics contribute to the higher levels of grit we observed in our OI cohort, and also whether these results are both reproducible and consistent in larger numbers of people with OI including more OI subtypes. There was also no difference in the level of pain catastrophizing seen in the OI cohort compared to the general population despite previous studies reporting that many individuals with OI are living with chronic pain [6, 7]. To the best of our knowledge, there are no previous studies on quality of life in OI which have sought to use these measures to understand the level of grit or pain catastrophizing experienced by individuals with OI. We believe that it is important for future studies to include these measures in order to better characterize how resilience and pain catastrophizing relate to other aspects of overall HRQoL.

The present study is not without its limitations. First of all, the study population primarily consists of those diagnosed with types I or IV OI, and only two participants with type III OI, which is the most severe, survivable form of OI by the Sillence classification. An individual’s clinical diagnosis does not always align with self-perceived OI severity, as many individuals with so-called mild OI can have significant disability, as well as face the impact of living with hidden disability. However, given the heterogeneous nature of OI, this skew in the cohort should be considered when drawing conclusions surrounding the health-related quality of life of the OI population as a whole. Future studies should include more individuals with type III OI to better characterize the relationship between different OI types and HRQoL. In addition, we would expect other factors such as aging and any comorbidities to play a role in individuals’ self-reported quality of life. Another limitation of this study is that while comparisons were made against previously published reference values from adults in the general U.S. population, it was not possible to closely match the normative data with the OI participant data in terms of age, sex, or socioeconomic status. Many of the normative studies were also published in the 1990s and early 2000s. These factors should all be taken into account when comparing these results, and future studies should be compared to modern normative data with ethnic and demographic diversity. Lastly, a challenge of this study is the spread of data and high standard deviations due to variation in participants’ individual survey responses. Overinterpretation of nonsignificant findings should be done with care given the limited sample size and exploratory nature of the present study. Follow-up studies should be conducted with larger numbers and a more balanced sample of participants, including more male participants and participants with severe, type III OI, which would allow for confirmatory regression analyses to corroborate these findings.

## Conclusions

In this study, we observed that adults with OI reported lower physical health scores but no difference in mental health scores compared to the general population. The outcomes of this prospective study demonstrate that individuals with OI are remarkably strong in that they have both high levels of grit and do not engage in pain catastrophizing behavior despite the considerable physical challenges associated with their condition. However, clinicians and healthcare professionals engaged with individuals with OI should still remain conscious of the humanistic burden of living with OI, defined by Schoser and colleagues as “the impact of an illness on a patient’s health-related quality of life (HRQoL), activities of daily living (ADL), caregiver health, and caregiver QoL.” [44]. Our findings suggest that individuals with OI may benefit from specialized therapy or interventions aimed at addressing and remedying their perceived day-to-day limitations, although future research is necessary in order to better understand the kinds of interventions that may be most successful in addressing the unique concerns of this population. Continued efforts towards building community for individuals with OI and their families, such as offering participation in online or in-person groups for connection with others living in similar circumstances, could play a significant role in alleviating the difference in social functioning reported by those with OI compared to the general population. Finally, investment should continue to be made in increasing awareness about OI within the healthcare community and the general public in order to promote understanding and garner support for individuals with OI. Above all, fostering a comprehensive approach that recognizes both physical and mental aspects of the diagnosis is paramount in providing effective long-term care and support to those diagnosed with OI.

## Data Availability

The data from the current study is available from the corresponding author on reasonable request.
